# Evaluation of a blocking ELISA for the detection of antibodies against *Lawsonia intracellularis *in pig sera

**DOI:** 10.1186/1751-0147-53-23

**Published:** 2011-04-01

**Authors:** Magdalena Jacobson, Per Wallgren, Ann Nordengrahn, Malik Merza, Ulf Emanuelson

**Affiliations:** 1 Department of Clinical Sciences, Faculty of Veterinary Medicine and Animal Husbandry, Swedish University of Agricultural Sciences, 750 07 Uppsala, Sweden; 2 National Veterinary Institute, 751 89 Uppsala, Sweden; 3 Svanova Biotech, 751 45 Uppsala, Sweden

## Abstract

**Background:**

*Lawsonia intracellularis *is a common cause of chronic diarrhoea and poor performance in young growing pigs. Diagnosis of this obligate intracellular bacterium is based on the demonstration of the microbe or microbial DNA in tissue specimens or faecal samples, or the demonstration of *L. intracellularis*-specific antibodies in sera. The aim of the present study was to evaluate a blocking ELISA in the detection of serum antibodies to *L. intracellularis*, by comparison to the previously widely used immunofluorescent antibody test (IFAT).

**Methods:**

Sera were collected from 176 pigs aged 8-12 weeks originating from 24 herds with or without problems with diarrhoea and poor performance in young growing pigs. Sera were analyzed by the blocking ELISA and by IFAT. Bayesian modelling techniques were used to account for the absence of a gold standard test and the results of the blocking ELISA was modelled against the IFAT test with a "2 dependent tests, 2 populations, no gold standard" model.

**Results:**

At the finally selected cut-off value of percent inhibition (PI) 35, the diagnostic sensitivity of the blocking ELISA was 72% and the diagnostic specificity was 93%. The positive predictive value was 0.82 and the negative predictive value was 0.89, at the observed prevalence of 33.5%.

**Conclusion:**

The sensitivity and specificity as evaluated by Bayesian statistic techniques differed from that previously reported. Properties of diagnostic tests may well vary between countries, laboratories and among populations of animals. In the absence of a true gold standard, the importance of validating new methods by appropriate statistical methods and with respect to the target population must be emphasized.

## Background

*Lawsonia intracellularis *is a common cause of chronic diarrhoea and poor performance in young growing pigs [1,2]. In some herds, the disease may also be manifested as severe haemorrhagic diarrhoea with high mortality rates [3]. Cultivation of this obligate intracellular bacterium is difficult and the diagnosis is therefore based on demonstration of the microbe or microbial DNA in tissue specimens or faecal samples by techniques such as PCR [4]. Further, the development of specific antibodies to *L. intracellularis *are monitored by serology and used, for instance, in the screening of herd prevalence and herd profiling.

Several serological methods have been described. The first method commercially available was an immunofluorescent antibody test, IFAT [5], with a stated sensitivity of 91% and specificity of 97% (Elanco Animal Health Indianapolis, Indiana, USA). This widely used method is based on the incubation of sera with semi-purified *L. intracellularis *antigen, and the results are visualised following incubation with anti-porcine fluorescein isothiocyanate conjugate. More recently a principally similar method, the immunoperoxidase monolayer assay (IPMA), has been developed [6]. The latter method is however only commercially available to a limited extent.

Furthermore, several enzyme-linked immunosorbent assays have been described. Such methods include indirect ELISAs employing whole-cell antigens [7,8], DOC -extracted antigens [9], or targeting *L. intracellularis*-specific antigenic epitopes such as LPS [10], LsaA [11], or the FliC protein [12]. The stated sensitivities vary from 67 to 99.5% and the specificities from 93 to 100%. None of these methods are however available for the use in other laboratories. A blocking ELISA for the detection of antibodies to *L. intracellularis *has been developed and is commercially available [13]. To ensure a high specificity, the blocking ELISA is a direct sandwich ELISA that is based on monoclonal antibody-coated wells for capture of cell-cultured antigen and utilizes peroxidase-conjugated monoclonal antibodies as competitive antibodies. Several studies on prevalence and epidemiology of *L. intracellularis *have been performed employing this ELISA but the results have only been presented in conference proceedings. The method is reported to have a sensitivity of 96.5% and specificity of 98.7% with less than 10% coefficient of variation [13].

However, properties of diagnostic tests may well vary among populations of animals [14]. Therefore, they should always be validated in the target population and not solely in experimental animals [15], which is most commonly done. There is currently no scientific publication available on the evaluation of the blocking ELISA. Estimating the diagnostic sensitivity and specificity requires that a true gold standard is available. However, although the IFAT has been frequently used as a reference test, it cannot be considered to be a gold standard test. Still, validation is possible even without a true gold standard, but it require that appropriate statistical methods should be used to correct the estimates of sensitivity and specificity of the new test with respect to the imperfect reference test [15].

The aim of the present study was to evaluate the blocking ELISA in the detection of serum antibodies to *L. intracellularis*, by comparison to the previously widely used IFAT. Bayesian modelling techniques were used to account for the absence of a gold standard test. In addition, the current infection status of the animals was also estimated by other techniques.

## Methods

The study was approved by the Ethical Committee for Animal Experiments, Uppsala, Sweden.

### Herds and animals

Altogether, 176 pigs from 24 herds were included. Sera had been collected during two previous studies, A [1] and B [16], and were stored at -80°C. The pigs were 8-12 weeks old and had not been treated with antibiotics during the last two weeks prior to sampling. In study A, 54 pigs from nine poor-performance herds with diarrhoea among growing pigs and 12 pigs from four herds with a good performance and no problems with diarrhoea were included. In study B, sera were obtained from eleven herds that were selected on the basis of an ongoing problem with diarrhoea among growing pigs. From each herd, ten pigs that were suffering from diarrhoea and poor performance were selected (in total 110 pigs).

### Sampling and analysis

Theoretically, the number of samples required validating an assay at 0.95 confidence levels with a 2% error allowed and an estimated diagnostic sensitivity (DSn) of 0.965 should be 324, and the number of samples required to obtain an estimated diagnostic specificity (DSp) of 0.987 should be 123. With a 3% error allowed, the number of samples would be 144 and 55, respectively [17].

In study A, the pigs were transported to the laboratory, stunned and exsanguinated, and a blood sample was collected. At necropsy, individual samples were analysed for the presence of *Lawsonia intracellularis, Brachyspira *spp,* Campylobacter *spp, *Clostridium perfringens*, *Escherichia coli, Salmonella *spp, *Yersinia *spp, parasites and rotavirus [1]. In study B, individual blood and rectal faecal samples were collected at the herd visit for analysis of serum antibodies to *L. intracellularis *and for *L. intracellularis *DNA by nested PCR, respectively [16].

Sera were analysed by the blocking ELISA (Product No. 461379, German registration number FLI-B 390, bioScreen European Veterinary Disease Management Center GmbH, D-481 49 Münster, Germany). Positive and negative control sera were supplied by the manufacturer. Test sera were added in duplicate, incubated for one hour at 37°C, washed three times and incubated with horseradish peroxidase-conjugated anti-*Lawsonia intracellularis *monoclonal antibody for one hour at 37°C. Following washing, substrate were added, incubated for 10 minutes at room temperature, stop solution was added and the resulting OD value was read within 15 minutes at 450 nm. As it is a blocking ELISA, a negative result would thus have a high absorbance value. The absorbance values were then converted to the calculated percent inhibition (PI) by the formula

Sera were also prepared by the IleiTest and analysed according to the manufacturer's instructions (Elanco Animal Health, Indianapolis, Indiana, USA). The slides were read in a fluorescence microscope at 470 nm in 100 × magnification (FL-filter, Zeiss/Axioskop 20, C. Zeiss Svenska AB, Stockholm, Sweden) and all samples were independently examined by three persons and judged as positive or negative with respect to the presence of antibodies to *L. intracellularis *by comparison to the positive and negative controls included.

### Statistical analysis

The estimation of the diagnostic sensitivity and specificity of the blocking ELISA was performed in WinBUGS version 1.4 [18] using Bayesian techniques as described by Branscum et al. The results of the blocking ELISA was modelled against the IFAT test with a "2 dependent tests, 2 populations, no gold standard" model [19]. WinBUGS version 1.4 code for models based on conditional dependence among pair of tests is available from http://www.epi.ucdavis.edu/diagnostictests. This method does not employ a gold standard but requires results from two tests in two populations that differ in the prevalence of the disease. In this analysis, animals in study A and study B were considered as the two populations.

The method also requires prior information about some of the unknown parameters, *e.g*. sensitivity and specificity of the tests. Such prior information is often specified either from published papers or expert's best guess and the uncertainty is modelled through the use of beta distributions [20]. The prior mode (most probable) value for the sensitivity of the ELISA was considered to be 0.96, and the 5th percentile 0.75, with a corresponding transformed distribution beta(a, b) of beta(13.44, 1.52). The specificity mode prior for the ELISA was 0.98, the 5th percentile 0.75, with beta(11.74, 1.22). Corresponding values for the sensitivity of the IFAT were 0.85, 0.30 and beta(2.86, 1.33), and for the specificity 0.95, 0.30 and beta(2.59, 1.08), *i.e*. rather diffuse distributions [21]. The beta prior distributions for the prevalence in the two populations were also diffuse and equal for the two populations with a mode of 0.60, a 5th percentile of 0.30 and beta(4.84, 3.56). The beta prior distributions for the conditional probabilities (λDc and γDc) were the same as for the sensitivity and specificity, respectively, of the IFAT. These prior distributions were selected based on prior experiences gained from diagnostic work. The construction of prior distributions was done by the use of the Betabuster public domain software http://www.epi.ucdavis.edu/diagnostictests.

Estimates of sensitivity and specificity for the ELISA was first determined at PI-values ranging from 10 to 80 (by increments of 2), with results plotted in a two-graph receiver operating characteristic curve. Subsequently, estimates at PI-values 25 and 35 were also determined. All models were run with 100,000 iterations, where the initial 10,000 iterations were considered as burn-in and discarded from the evaluation.

## Results

### Herds and animals

In study A, *L. intracellularis *DNA was demonstrated by PCR in 29 of 54 animals from the poor performance herds. By IFAT, 47 sera from these pigs were judged as positive, while 37 and 34 sera were positive by the ELISA using cut-off levels of PI 30 and 35, respectively. *L. intracellularis *was not demonstrated in any of the pigs (n = 12) from the good performance herds, and by the ELISA, PI was <21 in all pigs, whereas three pigs were positive by IFAT. In study B, *L. intracellularis *DNA was demonstrated in 24 of 110 animals from 5 herds. According to the IFAT, 20 animals from 4 herds were seropositive. By employing a cut-off value of PI 30 in the ELISA, 46 animals from 9 herds were seropositive. Using a cut of value of PI 35, 25 animals from 5 herds were seropositive (Table 1).

**Table 1:** Positive results obtained by analysis of faeces and sera from 176 pigs in 24 herds collected during two previous studies, A and B, and analysed by nested PCR and by two serological methods, ELISA and IFAT.

	PCR	ELISA (PI > 30)*	ELISA (PI > 35)*	IFAT
**Study A**. Pigs from poor performance herds				
*No of positive animals (%)*	29 (44)	37 (56)	34 (52)	47 (71)
*No of positive herds (%)*	8 (89)	9 (100)	9 (100)	9 (100)
**Study A**. Pigs from good performance herds				
*No of positive animals (%)*	0	0	0	3 (25)
*No of positive herds (%)*	0	0	0	3 (75)
**Study B**. Pigs with diarrhoea and poor performance				
*No of positive animals (%)*	24 (22)	46 (42)	25 (23)	20 (18)
*No of positive herds (%)*	5 (45)	9 (82)	5 (45)	4 (36)

*In the blocking ELISA, two cut-off values (PI 30 and PI 35, respectively) were evaluated.

### Statistical analysis

**Figure 1:**
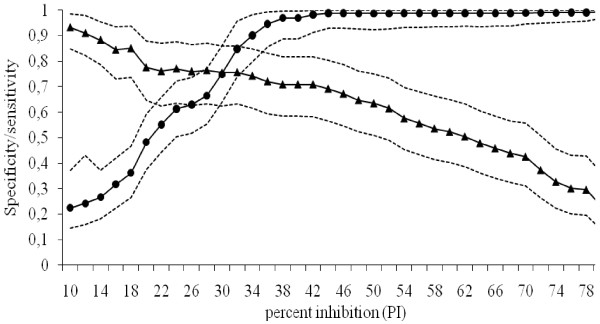
**The two-graph receiver operating characteristics curve, with cut-off values for the blocking ELISA test given as percent inhibition (PI) and ranging from PI 10 to 80 (by increments of 2)**. Sensitivity (black triangle) and specificity (black dot) for the ELISA were estimated by the use Bayesian modelling techniques. The intersection of the lines corresponds to the cut-off value for the ELISA test recommended by the manufacturer, PI 30. The 95% probability intervals are given by the dotted lines.

The two-graph receiver operating characteristics curve, with cut-off values for the ELISA test ranging from 10 to 80, is presented in Figure 1. The intersection of the lines corresponds to the cut-off value for the ELISA test recommended by the manufacturer, *i.e*. PI 30. The median of the posterior estimates of the sensitivity and specificity of the ELISA test was 0.76 and 0.62, respectively, at a cut-off of PI 25 with the 95% probability intervals 0.63 - 0.87 and 0.51 - 0.73, respectively. Employing a cut-off value of PI 30, the estimated sensitivity and specificity was 0.75, with 95% probability intervals 0.63 - 0.86 and 0.64 - 0.86, respectively, and employing a cut-off value of PI 35, the estimated sensitivity was 0.72 and the specificity was 0.93, with 95% probability intervals 0.59 - 0.83 and 0.83 - 0.99, respectively. The median of the posterior estimate for the sensitivity of the IFAT was 88% and the specificity was 95%. The estimates were very stable irrespective of the cut-off used for the ELISA test.

Using PI 30 as cut-off, the IFAT and the blocking ELISA had an observed agreement of 70%. Using PI 35 as cut-off, the agreement was 80% (Table 2). The positive predictive value, *i.e*. the probability of disease given a positive test result, would be 0.82 at a cut-off of PI 35 (sensitivity 0.72, specificity 0.93) and an estimated prevalence of 30%. The corresponding negative predictive value would be 0.89.

**Table 2:** The number of positive (+) and negative (-) results in the analysis of antibodies to *L. intracellularis *in 176 sera obtained from growing pigs with or without diarrhoea and analysed by two different methods, an immunofluorescent antibody test, IFAT, and a blocking ELISA.

		IFAT	
		
		+	-
ELISA (PI >30)*	**+**	49	34
	**-**	18	75
ELISA (PI >35)*	**+**	45	14
	**-**	22	95

*In the blocking ELISA, two cut-off values (PI 30 and PI 35, respectively) were evaluated.

## Discussion

To assess the performance of a new method, the diagnostic sensitivity (DSn) and specificity (DSp) should be determined in reference samples with known history and infection status [8,9,22]. However, these samples may not reflect the actual population for which the methods are intended and thus, the accuracy of the test might vary. In the present study, Dsn and Dsp of the ELISA applied to samples obtained from the target population differed from the previously established values. To cope with the problem of having an imperfect gold standard, Bayesian estimation was applied as recommended by OIE. The Bayesian estimation is a latent class model that does not assume the previously used method to be the best method (*i.e*. used as gold standard) and the known sensitivity and specificity of the reference test are not a prerequisite.

To further illustrate the importance of evaluating the method in the target population with a "natural" distribution of positive and negative animals, reference samples from two populations with known disease status (animals that were confirmed to be infected with *L. intracellularis *by PCR on faecal samples and animals from a high health herd where *L. intracellularis *had not been demonstrated) were analysed by both methods and compared by a 2 × 2 table using the IFAT as gold standard. This resulted in 100% specificity and sensitivity for both methods employed (data not shown).

Several cross-sectional studies on the serological prevalence of *L. intracellularis *in Europe and Asia have been conducted using the blocking ELISA but very few studies have attempted to validate the method. Keller et al. (2006) reported a good reproducibility between different laboratories. Further, in comparison to IFAT, the ELISA provided a higher sensitivity and more unambiguous results. By comparison to an in-house IFA, the DSn and DSp were estimated to 92 and 98%, respectively [23]. However, these results have only been presented in conference proceedings. In previous validations of the IFAT, the DSn varied from 58 to 90% and the DSp from 92 to 100% [5,21,24].

The results partly reflect the difficulties to clinically assess the stage of infection. Based on previous results, >40% of the pigs aged 9 to 15 weeks with clinical signs indicative of proliferative enteropathy were expected to shed the microbe in faeces [25,26]. Experimentally, pigs developed diarrhoea 7-14 days post inoculation in a dose-dependent manner [27], and circulating *L. intracellularis*-specific antibodies was first detected 2 weeks after challenge [28]. The purpose of study A was to determine the primary cause of diarrhoea in growing pigs and therefore, pigs were sampled immediately at the commencement of clinical signs. Hence, only a few pigs were expected to have seroconverted. However, most pigs turned out to be seropositive and slowly progressing infections will probably remain subclinical for some time before being detected [29]. Study B targeted growing pigs with diarrhoea, irrespective of the commencement of clinical signs and therefore, most pigs were expected to have seroconverted [16]. Furthermore, some animals will not seroconvert in response to infection [3,26].

Several other methods are reported to have a good performance with high DSn and DSp [8,9,22]. However, these methods use various gold standards and target populations and the results given in previous papers are therefore not possible to use for comparison. Further, they are presently not commercially available. Hence, sera must be submitted to these particular laboratories and factors such as geographical variation and local variations in infectious load may not be accounted for. Only the two methods employed in the present study are commercially available and may be adapted to various laboratories. The IFAT have been widely used, but the method is laborious and time-consuming, and discrepancies in interpretation of the results between various laboratories have been reported [30].

On the other hand, several studies report a high prevalence (90-100%) of seropositive finisher pigs close to slaughter (25-27 weeks of age), despite that shedding of *L. intracellularis *or chronic intestinal lesions are rarely reported [9,21]. This may be caused by booster infections [9], but since shedding in growing pigs may occur at short intervals [21,26], and circulating antibodies may be detected for 5 weeks only [3,21], the consistently high levels of antibody found in older pigs remains to be clarified. The blocking ELISA is based on monoclonal antibodies for capture and blocking to ensure a defined analytic sensitivity and specificity and, assumingly, an increased diagnostic specificity. However, if antibodies are formed towards other antigens with similar antigenic epitopes, unspecific or crossreactivity reactions may occur [24]. This was however not investigated in the present study.

In conclusion, the diagnostic sensitivity of the blocking ELISA was 72% and the diagnostic specificity was 93%, as evaluated by Bayesian statistic techniques. This technique allows the validation of a diagnostic method also without a true gold standard. The positive predictive value was 0.82 and the negative predictive value was 0.89, when the analysis was applied on samples from growing pigs in the age of 8-12 weeks. The sensitivity and specificity demonstrated in the present study differed from that previously reported and the importance of validating new methods with respect to the target population was emphasised.

## Competing interests

The authors declare that they have no competing interests.

## Authors' contributions

MJ, PW, AN and MM participated in the design of the study. MJ were responsible for the coordination of the work, for collecting the samples, for the PCR analyses and drafted the manuscript. PW were responsible for the serological analyses. UE were responsible for the statistical analyses and drafted this part of the manuscript. All the authors red and approved the manuscript.
